# Predicting preterm birth using auto-ML frameworks: a large observational study using electronic inpatient discharge data

**DOI:** 10.3389/fped.2024.1330420

**Published:** 2024-01-31

**Authors:** Deming Kong, Ye Tao, Haiyan Xiao, Huini Xiong, Weizhong Wei, Miao Cai

**Affiliations:** ^1^ Wuhan Children’s Hospital (Wuhan Maternal and Child Healthcare Hospital), Tongji Medical College, Huazhong University of Science and Technology, Wuhan, Hubei, China; ^2^ Department of Epidemiology, School of Public Health, Sun Yat-sen University, Guangzhou, Guangdong, China

**Keywords:** preterm birth, machine learning, administrative data, China, autoML

## Abstract

**Background:**

To develop and compare different AutoML frameworks and machine learning models to predict premature birth.

**Methods:**

The study used a large electronic medical record database to include 715,962 participants who had the principal diagnosis code of childbirth. Three Automatic Machine Learning (AutoML) were used to construct machine learning models including tree-based models, ensembled models, and deep neural networks on the training sample (*N* = 536,971). The area under the curve (AUC) and training times were used to assess the performance of the prediction models, and feature importance was computed via permutation-shuffling.

**Results:**

The H2O AutoML framework had the highest median AUC of 0.846, followed by AutoGluon (median AUC: 0.840) and Auto-sklearn (median AUC: 0.820), and the median training time was the lowest for H2O AutoML (0.14 min), followed by AutoGluon (0.16 min) and Auto-sklearn (4.33 min). Among different types of machine learning models, the Gradient Boosting Machines (GBM) or Extreme Gradient Boosting (XGBoost), stacked ensemble, and random forrest models had better predictive performance, with median AUC scores being 0.846, 0.846, and 0.842, respectively. Important features related to preterm birth included premature rupture of membrane (PROM), incompetent cervix, occupation, and preeclampsia.

**Conclusions:**

Our study highlights the potential of machine learning models in predicting the risk of preterm birth using readily available electronic medical record data, which have significant implications for improving prenatal care and outcomes.

## Introduction

Preterm birth, defined as delivery before 37 weeks of gestation, is a major public health challenge that affects around 15 million babies worldwide each year (1). It is a leading cause of neonatal mortality and morbidity, as well as long-term health problems such as neurodevelopmental disabilities and chronic diseases (2). The causes of preterm birth are multifactorial and complex, and include maternal factors such as infections, stress, and chronic diseases, as well as fetal and environmental factors (3). Despite efforts to reduce the incidence of preterm birth, the rate has not significantly decreased in recent years. This highlights the need for a better understanding of the underlying mechanisms and risk factors, as well as improved prevention and management strategies.

Health service and outcome research utilizing administrative medical databases is becoming increasingly popular and gaining more attention (4–9). Administrative data are routinely gathered from various healthcare institutions such as hospitals, clinics, and pharmacies. These data are extensive and provide comprehensive service utilization information, which has led to a surge in researchers using them for cost-effectiveness analysis, risk adjustment, and mortality and health outcome prediction. Prior research primarily uses traditional statistical models such as generalized linear models to construction prediction models, which fails to capture the nonlinear and complex relationships between potential risk factors and the outcome, and this bottleneck in model predictive performance may overshadow potentially important risk factors or bias the importance of each factor.

With the recent advancements in artificial intelligence and the associated applications in the medical field, machine learning models are closer to generate innovative solutions for healthcare than ever. For example, a study utilizing electronic medical records from Vanderbilt Hospital in the United States, highlighted the substantial advantages of machine learning in improving healthcare throughout the prenatal period via its superior performance in accuracy, classification, and portability (10). Additionally, a recently published review comprehensively examined the role of artificial intelligent as a promising tool for clinicians facing daily obstetric challenges (11). The review highlighted the potential of advanced machine learning algorithms to analyze vast amounts of medical data, aiding in the identification of new risk factors associated with premature birth. However, in real world practice, there is limited research on constructing powerful machine learning models for administrative medical data to predict premature birth.

Several challenges, including feature engineering, hyperparameter tuning, model specification, and model evaluation, hinder the hands-on applications of accurate machine learning models in the medical research. Automatic machine learning (AutoML) aims to address these challenges and generate machine learning products by automating algorithm selection, hyperparameter tuning, model evaluation, and others (12). In this study, we utilized three open-source AutoML frameworks (AutoGluon, Auto-sklearn, and H2O) to predict preterm birth based on data from 715,962 women who were hospitalized for childbirth in China.

### Related work

The existing research primarily focuses on using machine learning models for preterm birth prediction has significantly advanced our understanding in this field. Machine learning is a subdivision of artificial intelligence, possesses the capability to forecast patient clinical outcomes by extracting valuable features or predictor variables from data, and has been increasingly used in the prediction of preterm birth (13). For several decades, many researchers have used popular machine learning algorithms, including Support Vector Machine, K-Nearest Neighbors, and Convolutional Neural Networks. For instance, a study employed ordinary logistic regression, random forest, and KNN to identify risk factors for preterm birth in the USA. The study highlighted that a history of prior stillbirth, hypertension, and diabetes mellitus were significant risk factors for preterm birth (14).

A major limitation of these machine learning applications in preterm birth prediction is that they only smaller datasets (15) and this is partially from a lack of machine learning applications on electronic health records, which encompass millions of medical records, diagnoses, prescriptions, along with high-dimensional medical images (16). This study analyzes a large-scale electronic medical record database with 715,962 participants, which maximizes the statistical power to identify the risk factors associated with preterm birth in more depth, providing a scientific basis for evidence-based decision-making and guidelines.

Another notable limitation in earlier studies is the lack of applications on the vast repository of high-dimensional medical data available within electronic medical records. The current research acknowledges the untapped potential of these datasets and actively harnesses their breadth and depth to enhance the predictive modeling of preterm birth, enabling machine learning practitioners to derive meaningful insights and develop precise guidelines.

A common trend in previous studies involves heavy reliance on expert input for feature engineering, hyperparameter tuning, and model specification. This reliance can introduce subjectivity and make it challenging to reproduce superior performance. In contrast, the present study adopts a more automated approach. By leveraging three open-source AutoML frameworks—AutoGluon, Auto-sklearn, and H2O AutoML—the research automates critical steps such as algorithm selection, hyperparameter tuning, and model evaluation (17). The automation implemented in our study improves the reproducibility and scalability of the predictive models by boosting efficiency and reducing the reliance on extensive expert-defined phenotyping and ad-hoc feature engineering.

## Methods

### Data sources

This study used an administrative inpatient discharge dataset collected by the Health Commission of Shanxi Province, China from January 1, 2014 to December 31, 2017 (4–9). This database routinely collects data on individual's demographic information (age, sex, and ethnicity), socioeconomic status (marital status and occupation), disease severity, one main diagnosis code and up to 10 secondary diagnosis codes based on International Classification of Diseases, Tenth Revision (ICD-10). All female individuals with the main ICD-10 diagnosis code for pregnancy and childbirth (starting with “O”) were included in the study (18), yielding a final analytic sample of 715,962 participants. All individual and hospital identifiers, such as individual name, ID card number, and insurance card number, were excluded before the study team had access to the data. Observations with any missing data on the predictors were excluded from the study.

### Outcomes

Preterm birth was defined as a binary variable, with the value of one assigned to preterm birth that occurred before 37 weeks of gestation (identified by ICD-10 code O60.1) was coded as one, while the value of zero assigned to all other births (19).

### Predicting variables

We selected a few predicting variables (features) based on preexisting studies and data availability (20–23). Age was computed as the difference between the date of hospitalization and the date of birth. We coded ethnicity as Han Chinese and non-Han Chinese (minorities), and biological sex as male or female. Socioeconomic status was assessed through two multi-category variables: marital status (married, unmarried, widowed, divorced, and other) and occupation (public sector, private sector, agriculture, unemployed, and other) (24). Admission status was classified as normal, emergent, or dangerous, while admission source was categorized as inpatient admission, emergency department transfer, outpatient department transfer, or transfer from other medical facilities. Payment method included the New Rural Cooperative Medical Scheme (NRCMS), the Urban Employee Basic Medical Insurance (UEBMI), the Urban Resident Basic Medical Insurance (URBMI), self-payment, or others. We also identified a set of clinical risk factors for preterm birth, including gestational diabetes, premature rupture of membranes, preeclampsia, incompetent cervix, and nuchal cord. The ICD-10 clinical diagnosis codes for these risk factors are listed in Supplementary Table S1.

### Automl frameworks

AutoML can automate the process of algorithm optimization, hyperparameter tuning, model iteration, and model evaluation, and reduce the difficulty and time-consuming bottlenecks of machine learning models. We considered several popular AutoML frameworks for supervised machine learning. Auto-WEKA ceased development in March 2022. Auto-kera and Auto-PyTorch primarily focus on image pattern recognition and lack sufficient support for tabular classification data and its model evaluation metrics. Therefore, we did not use these three tools and this study chose three popular AutoML frameworks (AutoGluon, Auto-sklearn, and H2O AutoML).

#### Autogluon

A robust and accurate AutoML framework for structural data sets (25). We used AutoGluon-Tabular for binary classification for structural tabular dataset. It supports Graphics Processing Unit (GPU) training for most of its models including lightGBM, CatBoost, XGBoost, MXNet and FastAI Neural Networks (26–28). AutoGluon framework for tabular data, also known as AutoGluon-Tabular, is designed to train highly accurate machine learning models via ensembling multiple models and stacking these models in multiple layers (25). Therefore, the models implemented in AutoGluon are not generic models such as generalized linear models. The philosophy of AutoGluon is to train a curated list of most powerful models within a reasonable amount of time, so only around 10 complex ensembling models are implemented. Less performant generic models such as generalized linear models are not fitted in AutoGluon and can be implemented in other autoML frameworks. Instead of focusing on combined algorithm selection and hyperparameter optimization (CASH) problem, AutoGluon replies more on advanced data processing, deep learning, and multi-layer stack ensembling to maximize prediction performance (25).

#### Auto-sklearn

Auto-sklearn is an AutoML tool using meta-learning (29). The aim of meta-learning is to search for a better start for hyperparameter tuning using efficient and parameterized Bayesian optimization method, so that the initial start-off values are better than random. It is based on an existing Python machine learning package scikit-learn and does not support GPU acceleration in the current version. This framework so far supports 15 classifiers, 14 methods for feature preprocessing, and 4 data preprocessing methods, resulting in a total of over 100 hyperparameter combinations.

#### H2O AutoML

H2O AutoML is an open-source, highly scalable, fully automated, and distributed supervised learning algorithm set based on Java (30). The H2O AutoML tools includes hundreds of efficient supervised machine learning algorithms such as generalized linear models (GLMs), XGBoost, GBM (Gradient Boosting Machine), random forest, GBMs, and deep neural nets. It does not support GPU acceleration except for XGBoost models. The H2O AutoML also introduces a convenient tool of leaderboard to allow users to compare the predictive performance, including k-fold cross-validation, across models fitted by H2O AutoML framework.

Table 1 presents the number of machine learning models by model type and automatic machine learning frameworks. In this study, we only select the best 10 models (based on model evaluation metrics) for each model type to reduce the imbalance number of models per framework. Auto-sklearn fits the largest number of models (*N* = 60) among the three frameworks, followed by H2O AutoML (*N* = 33) and AutoGluon (*N* = 12).

**Table 1:** The number of machine learning models by model type and automatic machine learning frameworks.

	Auto-sklearn	AutoGluon	H2O AutoML	Mean	Total
Deep learning	10	2	10	7.33	22
GBM/XGBoost	10	3	10	7.67	23
KNN	10	2	0	4.00	12
LDA	10	0	0	3.33	10
Naive bayes	10	0	0	3.33	10
Random forest/trees	10	4	2	5.33	16
Stacked ensemble	0	1	10	3.67	11
GLM	0	0	1	0.33	1
Mean	7.5	1.5	4.1		
Total	60	12	33		105

GBM, gradient boosting machines; GLM, generalized linear models; KNN, K-nearest neighbors; LDA, linear discriminant analysis.

### Model evaluation metrics

To compare the model performance across different AutoML tools using a unified metric, we chose area under curve (AUC) for receiver operating characteristic curves as the model evaluation metric. The ROC curve depicts the relationship between the true positive and false positive rates while selecting cut-off values for predicting binary outcomes such as preterm birth (31). AUC, also known as the concordance statistic or c-statistics, is a measure of goodness of fit for binary classification models. It ranges between 0.5 and 1, where larger values indicate better prediction performance.

Model interpretability was evaluated as feature importance computed via permutation-shuffling (32). The feature importance scores measure the decrease in the predictive performance of a trained model when the values of a feature are randomly shuffled across rows. The features with high importance scores have the most significant contribution to prediction accuracy. These feature importance scores aid in interpreting the model's overall prediction performance by identifying the prioritized features it relies on for predictions.

### Computing environment and model setup

The original data (*N* = 715,962) were split into a training set (*N* = 536,971) and a test set (*N* = 178,991) based on the 75/25 criteria. We did not set up a validation data set as the hyperparameters were tuned using 10-fold cross validation on the training set. All the training, testing, and model evaluation were performed in a high-performance computing environment (96 threads and 512 GB RAM), with Python 3.9 installed on Linux CentOS 7.8 and one NVIDIA Tesla T4 enabled for GPU computing (CUDA version 11.7). The training was performed with a time limit of 24 h. The versions of the AutoML tools were AutoGluon (v0.7.0), Auto-sklearn (v0.15.0), and H2O AutoML (v3.40.0.2). The data and associated Python code to replicate the results can be found in Supplementary Materials.

## Results

### Descriptive statistics

Table 2 presents the descriptive features of patients, including their demographics, covariates, and health characteristics. In total, 715,962 participants were included in the study, with an average [standard deviation, (SD)] age of 28.4 (5.0) years. The overwhelming majority (99.9%) of the participants were of Han Chinese ethnicity. Of the total participants, 45,763 (6.39%) had a preterm birth, and they tended to be younger and have a higher prevalence of gestational diabetes, premature rupture of membranes, preeclampsia, and incompetent cervix.

**Table 2:** Overall participant characteristics and by preterm birth.

Variables	Overall	Preterm birth		
		No	Yes	*p*-value
	*N* = 715,962	*N* = 670,199 (93.61%)	*N* = 45,763 (6.39%)	
Age, mean (SD)	28.43 (5)	28.46 (5.01)	28.04 (4.83)	<0.001
Ethnicity, *N* (%)				0.362
Han	714,935 (99.86)	669,230 (99.86)	45,705 (99.87)	
Non-han	1,027 (0.14)	969 (0.14)	58 (0.13)	
Occupation				<0.001
Public sector	75,647 (10.57)	69,281 (10.34)	6,366 (13.91)	
Private sector	80,452 (11.24)	78,045 (11.65)	2,407 (5.26)	
Agriculture	253,496 (35.41)	236,779 (35.33)	16,717 (36.53)	
Unemployed	110,158 (15.39)	105,865 (15.80)	4,293 (9.38)	
Other	196,209 (27.41)	180,229 (26.89)	15,980 (34.92)	
Marital status, *N* (%)				
Married	675,328 (94.32)	631,716 (94.26)	43,612 (95.30)	<0.001
Unmarried	24,373 (3.40)	22,481 (3.35)	1,892 (4.13)	
Widowed	3,598 (0.50)	3,512 (0.52)	86 (0.19)	
Divorced	891 (0.12)	868 (0.13)	23 (0.05)	
Other	11,772 (1.64)	11,622 (1.73)	150 (0.33)	
Payment method, *N* (%)				<0.001
URBMI	51,228 (7.16)	49,314 (7.36)	1,914 (4.18)	
UEBMI	104,193 (14.55)	100,690 (15.02)	3,503 (7.65)	
NRCMS	304,449 (42.52)	293,644 (43.81)	10,805 (23.61)	
Self-payment	187,422 (26.18)	183,303 (27.35)	4,119 (9.00)	
Other	68,670 (9.59)	43,248 (6.45)	25,422 (55.55)	
Admission status, *N* (%)				<0.001
Normal	662,469 (92.53)	619,720 (92.47)	42,749 (93.41)	
Emergent	42,912 (5.99)	39,953 (5.96)	2,959 (6.47)	
Dangerous	10,581 (1.48)	10,526 (1.57)	55 (0.12)	
Admission source, *N* (%)				<0.001
Emergency department transfer	10,581 (1.48)	10,526 (1.57)	55 (0.12)	
Inpatient admission	12,640 (1.77)	12,533 (1.87)	107 (0.23)	
Transfer from other medical facilities	649,829 (90.76)	607,187 (90.60)	42,642 (93.18)	
Outpatient department transfer	42,912 (5.99)	39,953 (5.96)	2,959 (6.47)	
Gestational diabetes, N (%)				<0.001
No	710,780 (99.28)	666,244 (99.41)	44,536 (97.32)	
Yes	4,522 (0.63)	3,395 (0.59)	1,127 (2.68)	
Premature rupture of membranes, *N* (%)				<0.001
No	698,611 (97.58)	657,599 (98.12)	41,012 (89.62)	
Yes	17,351 (2.42)	12,600 (1.88)	4,751 (10.38)	
Preeclampsia, *N* (%)				<0.001
No	713,405 (99.64)	668,791 (99.79)	44,614 (97.49)	
Yes	2,557 (0.36)	1,408 (0.21)	1,149 (2.51)	
Nuchal cord, N (%)				<0.001
No	710,498 (99.24)	666,177 (99.40)	44,321 (96.85)	
Yes	5,464 (0.76)	4,022 (0.60)	1,442 (3.15)	
Incompetent cervix, *N* (%)				<0.001
No	704,349 (98.38)	662,156 (98.80)	42,193 (92.20)	
Yes	11,613 (1.62)	8,043 (1.20)	3,570 (7.80)	

SD, standard deviation; NRCMS, the new rural cooperative medical scheme; UEBMI, the urban employee basic medical insurance; URBMI, the urban resident basic medical insurance.

### Predictive performance of machine learning models

**Figure 1:**
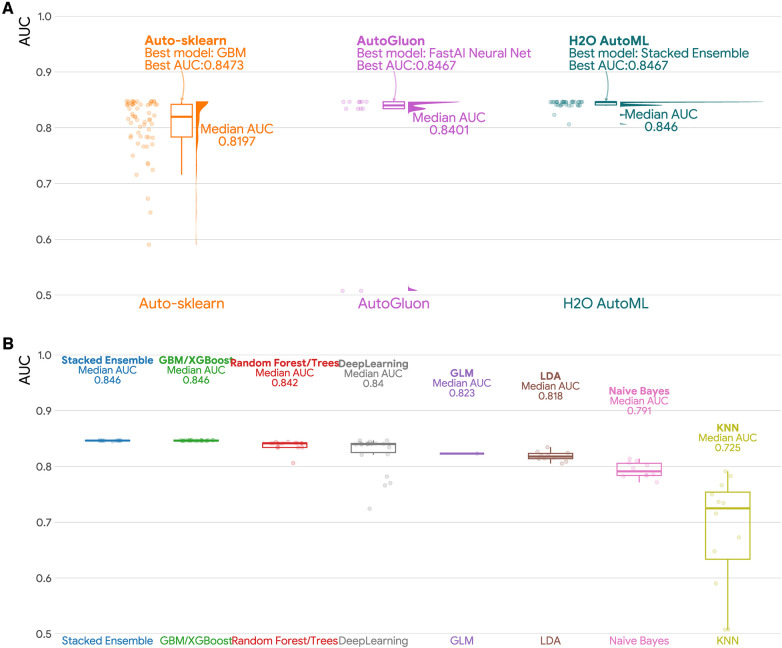
Area under the curve (AUC) for different AutoML frameworks and machine learning models. (**A**) Raincloud plot of the area under the curve (AUC) for three AutoML frameworks (Auto-sklearn, AutoGluon, and H2O AutoML). Each raincloud plot panel consists of three components: a jittered dot plot on the left side, a boxplot in the middle, and a cloud plot of the distribution of AUCs on the right side. (**B**) Boxplots of AUCs by machine learning models. GBM: Gradient Boosting Machines; GLM: Generalized Linear Models; KNN: K-Nearest Neighbors; LDA: Linear Discriminant Analysis.

To assess the predictive performance of machine learning models used in this study, we employed AUC as the evaluation metric. Results are presented in Figure 1 and Table 3, showing the AUC statistics for different models and AutoML frameworks. Our analyses revealed that GBM/XGBoost, Stacked Ensemble and random forrest/trees models performed better, with median AUC scores of 0.846, 0.846 and 0.842, respectively. Conversely, KNN, Naive Bayes, and LDA models achieved lower AUC scores with medians of 0.725, 0.791 and 0.818 respectively, which are even lower than a traditional logistic regression (AUC = 0.823). H2O AutoML had the best median predictive ability among the three frameworks, achieving a median AUC of 0.846, followed by AutoGluon (AUC = 0.840) and H2O AutoML (AUC = 0.820). It is notable that the maximum AUCs of the three H2O modeling frameworks were identical (AUC = 0.847).

**Table 3:** Summary statistics for area under the curve (AUC) for different autoML frameworks and machine learning models.

	No. of models	Mean	SD	Percentile				
				Min	25th	Median	75th	Max
Overall	105	0.814	0.062	0.508	0.809	0.841	0.846	0.847
A baseline logistic regression	1	0.823	–	0.823	0.823	0.823	0.823	0.823
By AutoML framework								
Auto-sklearn	60	0.804	0.053	0.59	0.784	0.82	0.842	0.847
AutoGluon	12	0.786	0.13	0.508	0.834	0.84	0.847	0.847
H2O AutoML	33	0.842	0.008	0.806	0.841	0.846	0.846	0.847
By machine learning models								
Deep Learning	22	0.825	0.033	0.724	0.825	0.84	0.841	0.847
GBM/XGBoost	23	0.847	0.001	0.846	0.846	0.846	0.847	0.847
KNN	12	0.684	0.101	0.508	0.634	0.725	0.754	0.791
LDA	10	0.818	0.009	0.805	0.814	0.818	0.823	0.835
Naive Bayes	10	0.794	0.014	0.772	0.784	0.791	0.806	0.814
Random forest/trees	16	0.838	0.009	0.806	0.834	0.842	0.842	0.844
Stacked ensemble	11	0.846	0	0.845	0.846	0.846	0.847	0.847
GLM	1	0.823	–	0.823	0.823	0.823	0.823	0.823

GBM, gradient boosting machines; GLM, generalized linear models; KNN, K-nearest neighbors; LD, linear discriminant analysis.

### Training time

**Figure 2:**
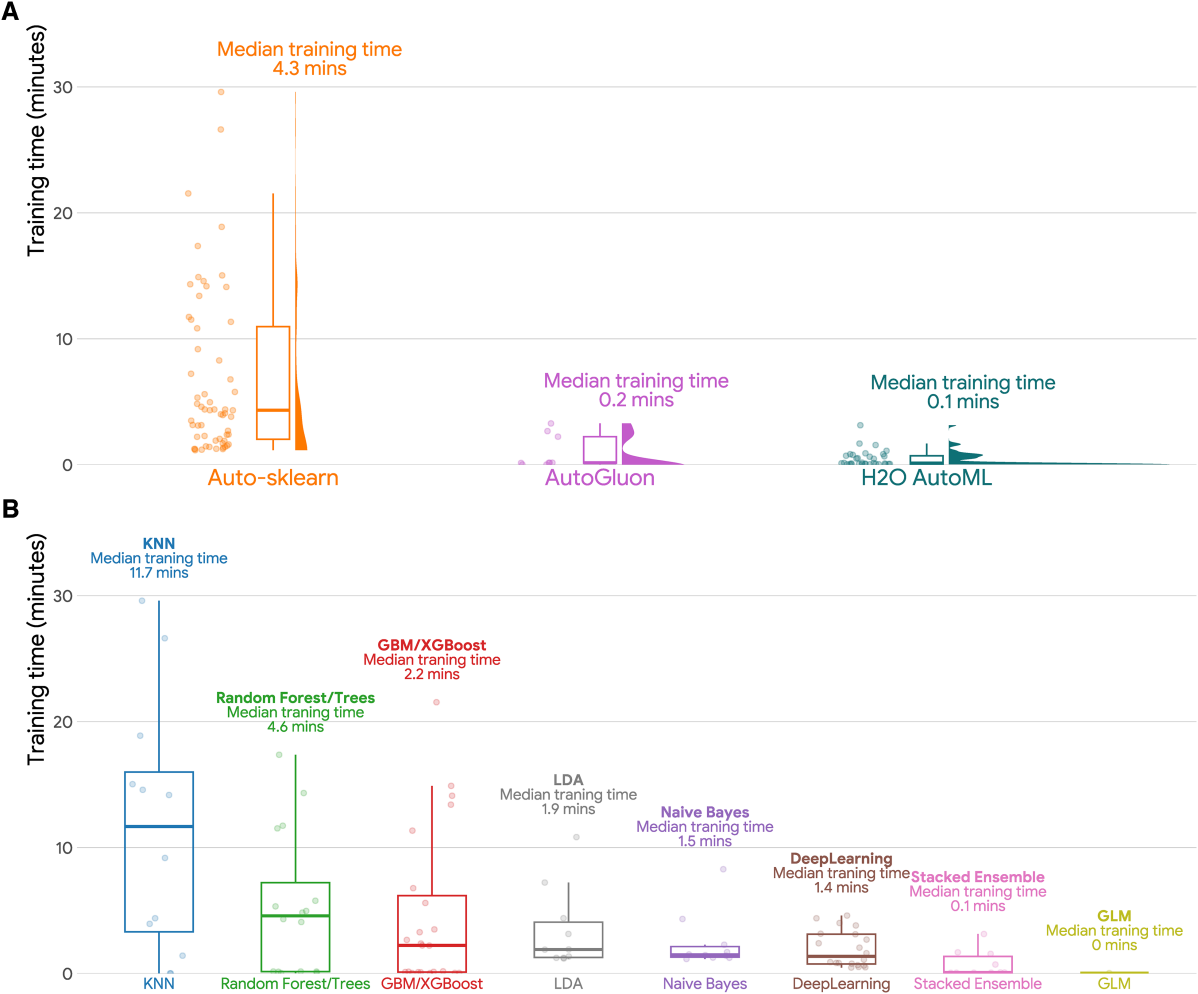
Training time in minutes for different AutoML frameworks and machine learning models. (**A**) Raincloud plot of training time in minutes (training set sample size *N* = 536,971) for three AutoML frameworks (Auto-sklearn, AutoGluon, and H2O AutoML). Each raincloud plot panel consists of three components: a jittered dot plot on the left side, a boxplot in the middle, and a cloud plot of the distribution of AUCs on the right side. (**B**) Boxplots of training time in minutes by machine learning models. GBM: Gradient Boosting Machines; GLM: Generalized Linear Models; KNN: K-Nearest Neighbors; LDA: Linear Discriminant Analysis.

Figure 2 and Table 4 shows that H2O AutoML had the lowest median training time (0.14 min) among the evaluated AutoML frameworks, followed by AutoGluon AutoML (median training time: 0.16 min) and Auto-sklearn (median training time: 4.33 min). Regarding machine learning models, GLM had the fastest training times, taking less time than Stacked Ensemble, Deep Learning, Naive Bayes, LDA, GBM/XGBoost, Random Forest/Trees and KNN.

**Table 4:** Summary statistics for training time in minutes for different autoML frameworks and machine learning models (training set sample size *N* = 536,971).

	No. of models	Mean	SD	Percentile				
				Min	25th	Median	75th	Max
Overall	105	4.25	5.93	0.01	0.48	1.8	4.55	29.6
By AutoML framework								
Auto-sklearn	60	6.81	6.59	1.15	2.02	4.33	10.96	29.6
AutoGluon	12	0.98	1.34	0.01	0.14	0.16	2.23	3.28
H2O AutoML	33	0.48	0.66	0.03	0.06	0.14	0.72	3.14
By machine learning models								
Deep Learning	22	1.96	1.45	0.47	0.75	1.36	3.13	4.6
GBM/XGBoost	23	4.56	6.17	0.04	0.11	2.23	6.19	21.54
KNN	12	11.49	10.08	0.01	3.31	11.68	16	29.6
LDA	10	3.44	3.23	1.2	1.27	1.91	4.08	10.83
Naive Bayes	10	2.47	2.25	1.15	1.3	1.49	2.14	8.28
Random Forest/Trees	16	5.31	5.61	0.03	0.16	4.58	7.22	17.37
Stacked Ensemble	11	0.75	1.06	0.06	0.06	0.08	1.35	3.14
GLM	1	0.03	-	0.03	0.03	0.03	0.03	0.25

GBM, gradient boosting machines; GLM, generalized linear models; KNN, K-nearest neighbors; LDA, linear discriminant analysis.

Overall, when it comes to selecting an AutoML framework or machine learning model, the training time is an important factor to consider. The choice of framework or model should be based on the specific requirements of the task at hand, including the available computational resources and the desired level of accuracy.

### Variable importance

**Figure 3:**
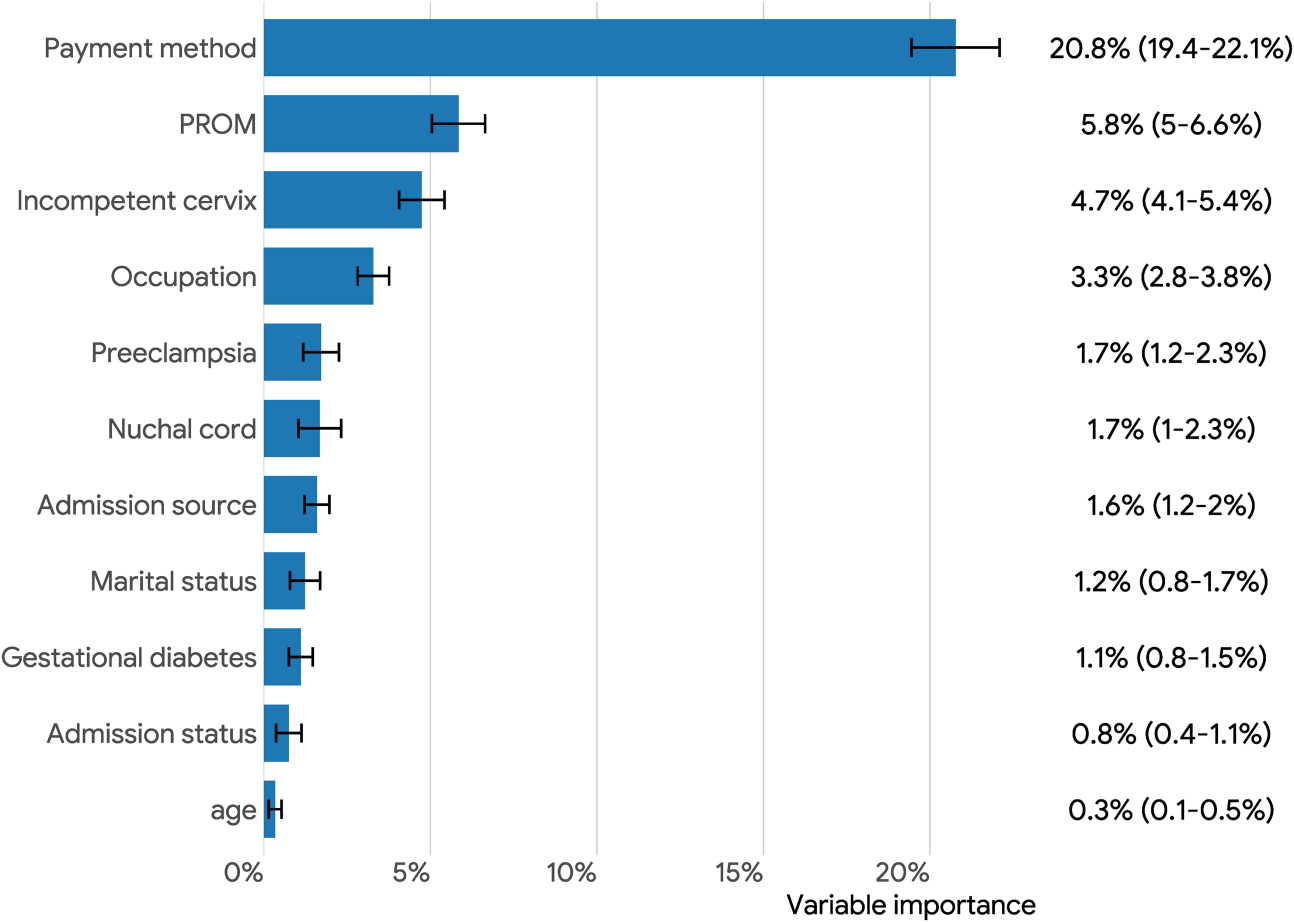
Overall feature importance (95% confidence intervals) plots for predicting preterm birth via permutation-shuffling in AutoGluon. PROM: premature rupture of membranes.

Figure 3 illustrates the important features for predicting preterm birth identified by AutoGluon frameworks. We utilized permutation feature importance to prioritize the variables by their importance in the FastAl Neural Net model. The top five variables were payment methods, premature rupture of membrane (PROM), incompetent cervix, occupation, and preeclampsia. These variables had a more substantial influence on the model's accuracy compared to the other variables in the dataset.

## Discussion

Preterm birth is the primary cause of neonatal death and disability, and early prediction of preterm birth has great potential to improve the survival rate of preterm infants (33). In this study, electronic medical record data from the hospital discharge database were selected to construct machine learning model to predict preterm birth. We found that payment methods, PROM, incompetent cervix, occupation, and preeclampsia were strongly associated with preterm birth patients.

AutoML has increasingly gain popularity since it substantially reduces the difficulty of building a machine learning pipeline via complex, iterative, and time-consuming technical details including hyperparameter tuning, algorithm selection, or model evaluation. All these processes are automated within a few lines of code, so it saves the effort and time of an expensive machine learning engineer. Our findings revealed that H2O AutoML, surpassing AutoGluon and Auto-sklearn, achieved reasonably well predictive performance within a short amount of time (median train time per model was 0.1 min for over half a million rows and 12 features).

This study confirmed that PROM and incompetent cervix are significant risk factors for preterm birth, consistent with previous research (22, 34). Several mechanisms may explain these associations. Intrauterine infection or inflammation is believed to be a major contributing factor to preterm birth PROM, subsequent preterm birth (35). The cervix is normally closed during pregnancy, but in cases of incompetent cervix, the cervix may dilate too early, triggering contractions and labor, which may lead to preterm birth (36).

The findings in our study show that payment method is associated with increased risk of preterm birth. The association between payment method and preterm birth is a complex issue. Research has revealed that socioeconomic status can be a contributing factor to preterm birth, with women who lack insurance or have public insurance being more susceptible to preterm birth compared to those with private insurance (37). One possible explanation for this correlation is that women with lower incomes may encounter more obstacles in accessing healthcare services, including prenatal care, which can elevate the risk of preterm birth. Additionally, women with public insurance may have limited choices in healthcare providers and receive less comprehensive care. However, it is important to note that the relationship between payment method and preterm birth is not entirely straightforward, and other factors, such as age, race/ethnicity, and marital status, may also play a role. More research is needed to comprehensively understand the complex factors.

We also observed the association between occupation and the risk of preterm birth. Specifically, public sectors are more susceptible to preterm birth than other occupations in China. One possible reason for this is that the job of a public sector is demanding and requires them to be constantly engaged in decision-making, multitasking, and dealing with high levels of stress. This pressure can take a toll on their mental health, leading to anxiety, depression, and other mental health issues, which can lead to preterm birth.

### Strengths and limitations

The main strength of this study is the large sample size of over 700,000 women in Shanxi province of China, which enhances the reliability and statistical power of the models conducted in this study. Additionally, we evaluate three metrics including the predictive performance, training times, and feature importance, which could give medical practitioners and machine learning engineers a more practical guidelines on choosing the most appropriate tool to work with. Third, we compared the results of different AutoML frameworks and machine learning models in this study, allowing us to precisely identify risk factors for preterm birth.

Several limitations should be noted. First, our study is an observational study that depends on secondary data, it is subject to potential information bias and residual confounding caused by inaccurate coding, hospital characteristics or unobserved patient. Second, a predominate proportion of the study participants were Han Chinese, thus the results cannot be generalized to populations with different ancestries. Third, since the three autoML frameworks were independently developed by different teams with different optimizing philosophies, the type and number of models implemented in each framework are not directly comparable.

## Conclusion

We employed several popular AutoML frameworks and machine learning to analyze a large Chinese electronic medical record database, assessing the risk factors associated with the risk of preterm birth. Our findings have the potential to screen high-risk populations for preterm birth in China, which can help doctors tailor treatments for pregnant women with different risks of preterm birth.

## Data Availability

The code and data supporting the conclusions of this article will be made available by the authors, without undue reservation.
