# Supplementary material for: Predicting preterm birth using auto-ML frameworks: a large observational study using electronic inpatient discharge data
Source: Front Pediatr. 2024 Jan 31;12:1330420. doi: 10.3389/fped.2024.1330420 (PMC10867966; doi:10.3389/fped.2024.1330420)
Supplement: Supplementary file 1 [file Table1.docx]

**Supplemental Materials**

**Table S1**. ICD-10 codes for clinical risk factors for preterm birth

| clinical risk factors | ICD-10 codes |
| --- | --- |
| Gestational diabetes | O24.4 |
| Gestational hypertension | O16 |
| Uterine fibroids | D25 |
| Preeclampsia | O14 |
| Endometriosis | N80.9 |
| Multifetal pregnancy | O31 |
| Polyhydramnios | O40 |
| Incompetent cervix | O34.3 |
| Antiphospholipid syndrome | D68.6 |
| Nuchal cord | O69.1 |
